# Rectal perforation following endorectal prostate MRI: an unexpected complication

**DOI:** 10.1186/s12894-020-00591-9

**Published:** 2020-03-17

**Authors:** I Hsu, Chia-Chen Lee, Ming-Jenn Chen

**Affiliations:** 1 Division of plastic surgery, Department of Surgery, Chi-Mei Medical Center, Tainan, Taiwan; 2 Department of surgery, Kaohsiung Medical University Hospital, Kaohsiung, Taiwan; 3 Department of Surgery, Chi-Mei Medical Center, No.901, Zhonghua Rd., Yongkang Dist., Tainan City, 710 Taiwan Republic of China; 4 Department of Sports Management, College of Leisure and Recreation Management, Chia Nan University of Pharmacy and Science, Tainan, Taiwan Republic of China

**Keywords:** Rectal perforation, Endorectal prostate MRI, Prostate cancer, Complication

## Abstract

**Background:**

Prostate cancer is a common cancer among men in developed countries. Prostate magnetic resonance imaging (MRI) has been widely employed for early diagnosis of prostate cancer and recommending a treatment plan. The incidence of rectal perforation during endorectal prostate MRI is rare and has never been reported before. Herein, we present a case of rectal perforation after a prostate MRI examination that was subjected to emergency surgical intervention because of the acute presentation of generalized peritonitis. Patients with systemic comorbidities are reportedly at greater risks of encountering colonoscopic perforation. Endorectal prostate MRI is a safe diagnostic modality, but inadequate lubrication of the endorectal coil or over-insufflation of the balloon during the procedure may also lead to serious complications such as hollow organ perforation. Early surgery will be necessary should peritoneal symptoms persist.

**Case presentation:**

In 2015, a 56-year-old man came to our ER due to acute abdominal pain after he finished his MRI exam. The exam indicated diffuse tenderness over his abdomen and at the ER, his abdominal CT (computerized tomography) was checked. The images revealed extraluminal air in the perirectal fat and the pneumoperitoneum. In response, exploratory laparotomy, simple closure of rectal perforation, and loop-S colostomy were performed and the patient was discharged 1 month after operation.

**Conclusions:**

Prostate MRI is a secure procedure with few complications. Clinicians must keep in mind the possibility of perforation when using ultrasound probe. Hollow organ perforation can result in serious morbidity or death. As a result, patients need to be informed of the complications of prostate MRI. When performing the procedure, clinicians must be cautioned about the potential problems for patients with high-anesthetic risk.

## Background

Endorectal prostate MRI has shown to be a safe and useful modality that provides better image resolution in assessing prostate tumor localization, volume estimation, and staging. It not only improves the early diagnosis of prostate cancer by transrectal–ultrasound guided biopsy and serum PSA blood test, but also facilitates subsequent therapeutic planning. In general, prostate MRI rarely poses risks to average patients. The risks of prostate MRI include excessive sedation, allergic reactions to contrast materials, and contrast-induced nephrogenic systemic fibrosis. While an endorectal coil is applied, a gentle and meticulous operative technique is always required to minimize the chance of iatrogenic complications. The incidence of rectal perforation has not been reported elsewhere. The etiologic factors may link to the systemic comorbidities of the individuals, whereas endorectal coil insertion and latex balloon insufflation during the procedure may also impose direct trauma to the rectum.

## Case presentation

**Fig. 1:**
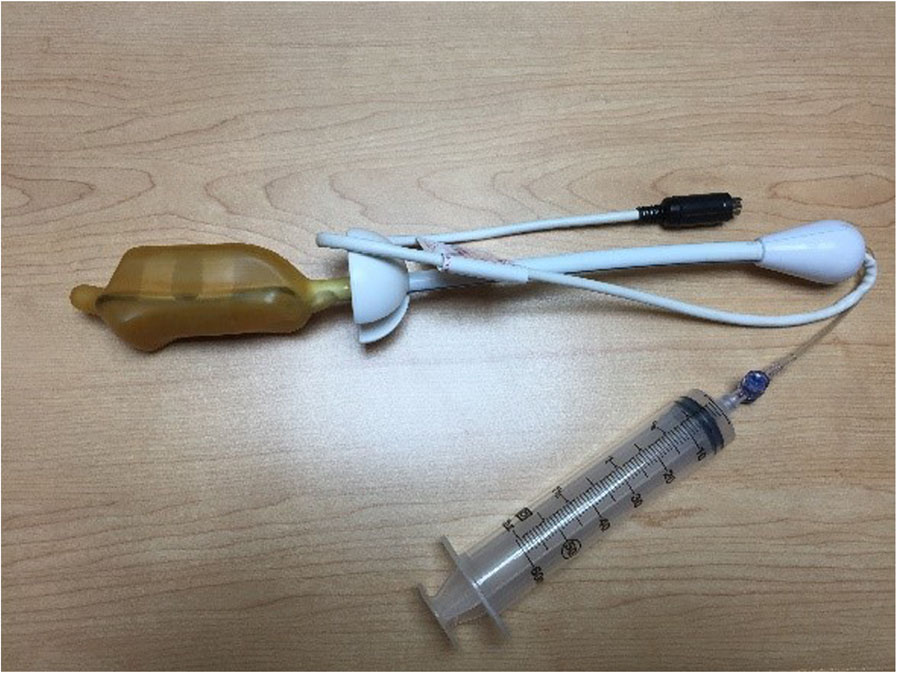
Medrad® Prostate eCoil MR Endorectal Coil. The probe improve your ability to visualize the internal architecture of the prostate and periprostatic structures, including prostate capsule and neurovascular bundles

**Fig. 2:**
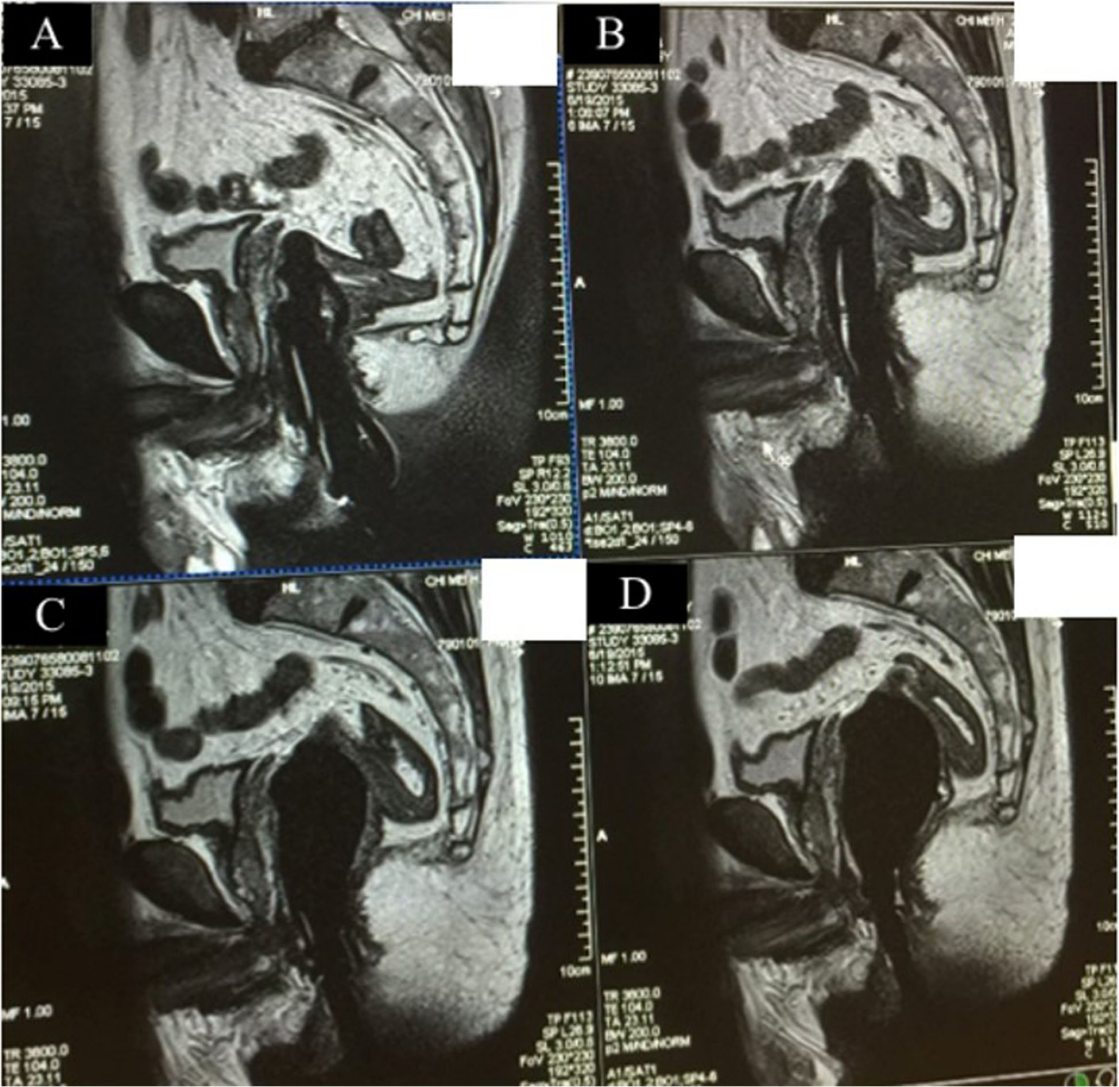
Balloon inflation with 30 ml (left) and 70–80 ml (right) of carbon dioxide, respectively

A 56-year-old man who had diabetes mellitus and a recent diagnosis of prostate cancer presented with acute abdomen to the emergency room about two hours after having received endorectal prostate MRI examination. Prior to the event, he had been initially referred to our hospital because of one-month history of gross hematuria. Serum prostate-specific antigen (PSA) was 3.99 ng per milliliter. Examination of transrectal ultrasound-guided (TRUS) biopsy specimen of the prostate revealed prostate adenocarcinoma in the right anterior and posterior aspect with Gleason score of 9 (4 + 5). A whole-body bone scan reported bone metastasis to the thoracic spine (T11). Therefore, endorectal prostate MRI was arranged to evaluate the extent of cancer for the best treatment path. Patient had a light diet 1 day before and undergone cleansing enema early morning at the hospital prior to the procedure, while metal and electronic objects were removed. A 1.5 T endorectal MRI/MRSI was used (Fig. 1). The endorectal coil was covered with latex condom to prevent contamination. During endorectal prostate MRI examination, patient maintained a left lateral decubitus position. The probe was lubricated with xylocaine jelly adequately and inserted through the anus to the rectum with mild force in cephalad fashion. Slight painful sensation of the lower abdomen was experienced momentarily but soon dissipated. The intrarectal balloon was inflated with 70 to 80 ml of carbon dioxide (Fig. 2) to reach maximal image resolution. Patient tolerated the procedure well without immediate complications and was sent home afterwards. The MRI result revealed advanced prostate cancer with transcapsular invasion to periprostatic tissue, right seminal vesicle and urinary bladder, metastatic regional lymphadenopathies and multiple bony metastases.

**Fig. 3:**
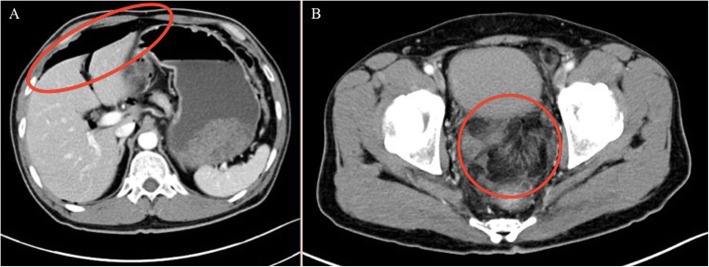
A supine abdominal CT showed diffuse pneumoperitoneum with mainly collected free-air around hepatic flexure (**a**) and perirectal extraluminal air with fat stranding (**b**)

**Fig. 4:**
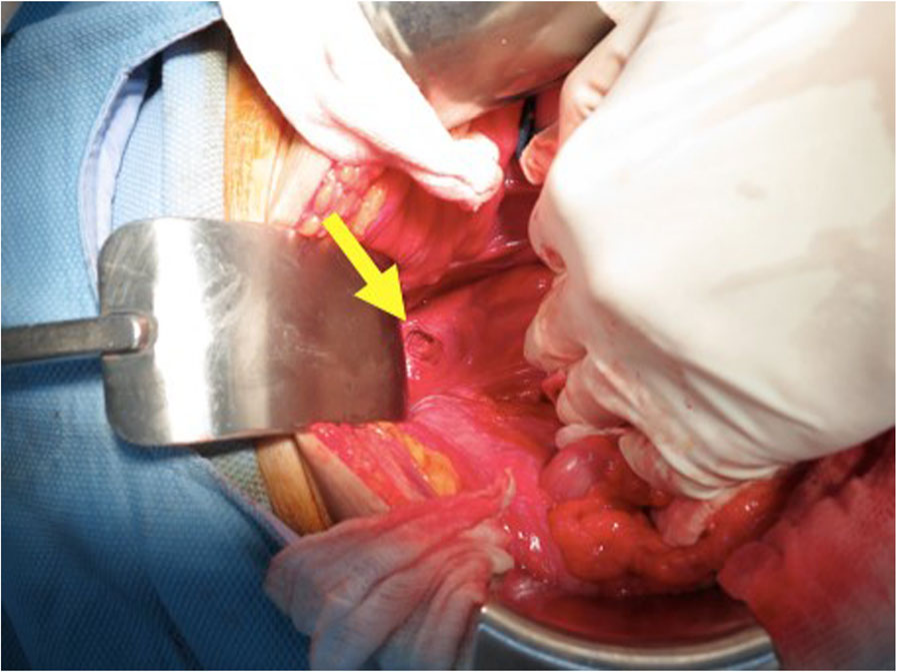
A rectal perforation, 2 cm in diameter, was observed in the anterior wall of the upper rectum (sharp arrow)

Approximately two hours following the examination, patient was seen in the emergency room because of a sudden onset of severe lower abdominal pain. A supine abdominal computed tomography (CT) showed diffuse pneumoperitoneum and perirectal extraluminal air with fat stranding (Fig. 3), prompting an emergency exploratory laparotomy. Intra-operatively, a rectal perforation about 2 cm in diameter at the anterior wall of the upper rectum was identified, suggesting an iatrogenic relation during endorectal prostate MRI examination (Fig. 4). A primary closure of the rectal perforation with sigmoid loop colostomy was performed. Nasogastric decompression was applied until the third day post-operatively. The intraoperative ascites culture result revealed ESBL-producing *Escherichia coli* infection. A concomitant urinary tract infection with *Pseudomonas aeruginosa* was reported. A colonoscopy was performed to evaluate the perforation site 10 days later. An approximately 8-mm residual perforation still presented in the anterior wall of the rectum, which was repaired by intraluminal endoclipping for perforation closure. The follow-up computed tomoraphy scan 20 days later showed absence of intra-abdominal free air. A good healing process of the perforation was seen during the colonoscopic examination.

## Discussion and conclusion

This is the first reported case of iatrogenic rectal perforation following endorectal prostate MRI. Endorectal prostate MRI is a safe modality that provides better image resolution for prostate cancer staging. Compared with other endoscopic procedures, the incidence of iatrogenic rectal perforation resulted from colonoscopic retroflexion was as low as 0.01% [1, 2], and such complication during endorectal prostate MRI examination is even rare. Despite the uncommonness, colonic or rectal perforations usually bring about serious mortality and morbidity. The most common site of colonic perforation is the rectosigmoid colon [2, 3]. In a 15-year study of Lüning et al. [ 4], most endoscopic perforations were diagnosed shortly after the procedure due to the presentation of acute abdomen as all perforations were subjected to surgery the same day. Patient with comorbidities of diabetes mellitus, chronic pulmonary disease, cardiovascular disease, cerebrovascular disease, liver or kidney diseases are at greater risks of encountering iatrogenic bowel perforations [2]. Gaetano et al. [5]reported a case of rectal perforation during defecography, causing extraluminal barium impaction, which successfully removed by TEM (Transanal Endoscopic Microsurgery). With the risk factors in mind, recognizing early signs of perforation, and directing early, and optimal treatment may reduce the chance of complications and death from iatrogenic colon or rectal perforations.

Endorectal prostate MRI is an excellent diagnostic tool for prostate cancer assessment. The mechanism of iatrogenic perforations during the procedure includes mechanical trauma from insufficient lubrication of the endorectal coil and barotrauma due to over-insufflation of the balloon. Meanwhile, we have to take into account the pre-existing morbidity of the patient. In our case, an experienced radiologist was in charge of performing endorectal insufflation technique during the examination, whilst there was no procedural abnormality encountered. However, the perforation injury might be related to the method of coil insertion and the distance of catheter insertion. Kayo Haruta et al. [6] pointed out that the distance between the anal verge and the rectum tends to increase with age as the maximum and minimum values for this distance differ for each age group. Rectal injury may occur at catheter insertion beyond 5.4 cm (sum of the anatomical length of the anal canal by 2.5 cm and the minimum measured distance by 2.9 cm) in the left lateral recumbent position. However, the best management of scope induced perforation remains controversial as the clinical condition and comorbidity of each patient should be considered. Patient of the case had metastatic prostate cancer, but the bowel was free from the involvement of prostate malignancy. Hence, tumor-related intestinal rupture was less likely. In addition, adequate bowel preparation during rectal probing of endorectal prostate MRI examination, good general health of patient, no underlying colonic pathology, absent or localized peritoneal sign, conservative management may be primarily considered before resorting to surgery [7]. If the vital signs are unstable or signs, surgical intervention will be necessary.

With an experienced operator, endorectal prostate MRI is a safe procedure with few complications. However, previous studies showed that high-anesthetic risk patients having a colonic perforation have relatively poor recovery and prognosis. Clinicians must be cautious about the possibility of iatrogenic perforation when introducing the endorectal coil and balloon inflation. Early diagnosis with appropriate management is the best strategy for patients with hollow organ perforation, which may progress to peritonitis or sepsis, resulting in serious morbidity or death. Hence, patients need to be informed of the complications of endorectal prostate MRI, and clinicians must be aware of the potential problems for patients with higher risk factors when performing the examination.

## Data Availability

The datasets used and/or analyzed during the current study are available from the corresponding author on reasonable request.

## Abbreviations

CT: Computerized tomography
MRI: Magnetic resonance imaging
PSA: Prostate-specific antigen
TEM: Transanal Endoscopic Microsurgery
TRUS: Transrectal ultrasound-guide
